# Dao Yin practice for student wellbeing: a classroom-based pilot study

**DOI:** 10.3389/fpsyg.2026.1821289

**Published:** 2026-05-12

**Authors:** Jian Zhao, Ziyu Zheng, Yu Li

**Affiliations:** 1 School of Arts, Taishan University, Tai’an, Shandong, China; 2 School of Music and Dance, Heze University, Heze, Shandong, China

**Keywords:** Dao Yin, mental health, mixed-methods, pilot study, psychologically vulnerable students, university students

## Abstract

**Background and objectives:**

University student mental health is a major public health and educational concern, yet preventive, low-threshold interventions embedded in routine academic settings remain limited. This study examined the feasibility, acceptability, and preliminary outcome signals of a Dao Yin-based dance/movement course for psychologically vulnerable Chinese undergraduates.

**Methods:**

This explanatory sequential mixed-methods pilot study was conducted from September 2024 to January 2025 at Taishan University, China. Thirty-eight students with elevated psychological distress completed a 16-session Dao Yin course delivered over 4 months. Quantitative outcomes included the Symptom Checklist-90 (SCL-90) total score and perceived physical status, which were analyzed using paired-samples t tests, 95% confidence intervals, and Cohen’s *d* effect sizes. Qualitative data were collected through focus groups and written narratives and analyzed using reflexive thematic analysis.

**Results:**

Pre-post analyses showed a significant reduction in SCL-90 total score and a significant improvement in perceived physical status. Effect sizes across symptom dimensions ranged from small to large. Qualitative themes highlighted bodily loosening, emotional settling, enhanced body–mind awareness, and the development of everyday self-regulation practices.

**Conclusion:**

A Dao Yin-based course delivered within the regular university curriculum appears feasible and acceptable for psychologically vulnerable students and showed preliminary positive signals for psychological and physical well-being. However, controlled studies are needed to establish efficacy. These findings provide initial support for integrating culturally grounded, body-oriented practices into campus mental health promotion.

## 1 Introduction

### 1.1 Mental health challenges among Chinese university students

University student mental health has become a priority public health concern globally. A substantial proportion of students worldwide experience clinically significant distress and functional impairment (Auerbach et al., 2018). In China, student mental health remains particularly worrisome and significantly affects academic performance. Sleep disturbances, depression, and other internalizing problems rank highest among concerns (Yu et al., 2021). A meta-analysis of Chinese mainland university students (2010–2020) identified sleep disorders, depression, and self-injurious behavior as the most prevalent problems, with anxiety, depression, and suicide attempts showing sustained upward trends throughout this decade (Chen et al., 2022). These patterns necessitate immediate preventive measures and enhanced campus support.

Psychological vulnerability among Chinese university students often stems from chronic stress exposure coupled with limited coping resources. Perceived stress is significantly associated with mental health status, while individual psychological resources moderate the stress–mental health relationship, indicating that students lacking protective resources may be particularly vulnerable when facing persistent academic and life stressors (Wang et al., 2024a). During the COVID-19 pandemic, meta-analyses again demonstrated widespread severe anxiety among Chinese university students, highlighting the urgent need for low-threshold psychological support services on campus (Wang et al., 2024b; Wang and Liu, 2022; Xu et al., 2022).

Among Chinese university students, poor sleep quality, fatigue, tension, and pain are common manifestations. Meta-analyses show sleep problems ranking as primary complaints (Chen et al., 2022; Yu et al., 2021). Recent evidence further suggests that anxiety, depression, sleep problems, and health-promoting lifestyles are closely interconnected among Chinese university students, indicating the need for integrated preventive approaches rather than isolated symptom-focused support (Sun et al., 2024). Interventions targeting somatic symptoms may simultaneously alleviate clinical and academic stress (Ryder et al., 2008). Qualitative research has revealed multiple barriers to psychological counseling among Chinese university students, including social stigma, confidentiality concerns, and unclear perceptions of service utility, which collectively contribute to low utilization of professional support (Gao et al., 2020; Ning et al., 2022). Help-seeking behavior is further influenced by perceived social norms and behavioral control (Pan et al., 2023). This underscores the importance of embedding low-threshold, non-stigmatizing support modalities within everyday campus life.

Consequently, Chinese educational authorities increasingly advocate integrating mental health support into regular instructional settings and developing preventive, accessible approaches embedded within daily academic activities rather than relying solely on professional psychological counseling. Grounded in this evidence base, the present study focuses on classroom-based, body-oriented approaches for psychologically vulnerable Chinese undergraduates, with particular attention to feasibility, acceptability, and preliminary signals of effect.

### 1.2 “Dao Yin” as a culturally grounded, body-oriented practice

“Dao Yin” (导引), literally meaning “guiding and pulling,” originated in ancient China as a body–mind cultivation practice documented in classical medical texts such as the Huangdi Neijing (Inner Canon of the Yellow Emperor). Unlike Tai Chi, which emphasizes martial arts-based choreographed sequences, or Qigong, which focuses primarily on static postures and qi cultivation, Dao Yin specifically targets the meridian system through stretching, twisting, and directing movement along energy pathways (Liu et al., 2021; Ma and Liu, 2020).

Dao Yin operates through three core principles that distinguish it as a systematically embodied practice: (1) “Guiding qi with form” (以形导气)—using physical movement to direct the flow of vital energy (qi) along specific meridians; (2) “Three adjustments” (三调)—the coordinated regulation of body posture (调身), breath (调息), and mental focus (调心); and (3) “Form-spirit unity” (形神合一)—achieving harmony between physical form (xing) and mental state (shen) through sustained internal awareness during movement. These principles position Dao Yin as a practice that cultivates physical balance and emotional harmony through regulated movement and interoceptive attention, rather than through aerobic exertion or cognitive restructuring.

In Western health psychology, body-oriented movement practices such as yoga, tai chi, and qigong have been extensively studied for mental health benefits. Yoga practice associates with reduced depression and anxiety through autonomic regulation and interoception (Cramer et al., 2013; Li et al., 2018). Tai chi and qigong demonstrate small-to-moderate improvements in psychological well-being, with proposed physiological mechanisms including reduced cortisol and enhanced vagal tone (Wang et al., 2010; Jahnke et al., 2010). While these practices share the characteristic of engaging the body as a regulatory system, they differ from Dao Yin in cultural origin and movement logic.

A notable gap exists in Dao Yin intervention research specifically targeting university student populations. Existing research has focused predominantly on physiological indicators among elderly or clinical populations (Zhou et al., 2019; Zhang and Chen, 2018), with limited attention to mental health effects among younger groups in educational settings. Most Dao Yin studies have conflated it with integrated protocols combining traditional Chinese medicine, meditation, or physical therapy, with few examining Dao Yin in isolation. Consequently, the distinctive psychological benefits of Dao Yin—particularly its emphasis on meridian-based stretching and form-spirit coordination—remain unclear.

Integrating Dao Yin principles into university classrooms may address the embodied dimensions of psychological distress among Chinese students, combining mental health education with culturally grounded, experiential learning rather than relying solely on cognitive or didactic modes. However, methodologically rigorous empirical research is urgently needed to examine the feasibility, acceptability, and preliminary signals of such interventions in authentic university environments.

### 1.3 Embodied cognition as theoretical framework

Embodied cognition theory posits that cognitive and affective processes are fundamentally grounded in bodily states, sensorimotor activity, and continuous interaction with the environment, rather than deriving solely from abstract neural computation (Macrine and Fugate, 2020). This theoretical framework contrasts sharply with traditional cognitivism, which conceptualizes the body as merely an input/output device for the brain. Instead, embodied cognition argues that bodily experiences constitute thinking and feeling themselves—body movement, posture, and sensory feedback are not secondary inputs but constitutive elements of perception, emotion, and meaning-making (Ye, 2010).

For example, Empirical evidence from Chinese action-idiom processing also supports the embodied nature of semantic understanding, suggesting that bodily experience and sensorimotor systems are involved in meaning-making (Su et al., 2013). When students adopt an upright, open posture during Dao Yin practice, they do not merely “think” about confidence or relaxation; the postural change itself actively shapes their affective state through bidirectional body–mind feedback loops. This “somatic marker” mechanism illustrates how physical sensations generate emotional signals that guide cognitive processing, explaining why movement-based interventions can directly influence psychological well-being without relying solely on verbal processing or cognitive restructuring.

Contemporary reviews emphasize that embodied processes play central roles in emotion regulation and adaptive behavior, particularly in contexts involving sustained bodily engagement (Castro-Alonso et al., 2024). Scholars advocating mind–body monism argue that bodily structure and movement patterns shape thinking, and therefore somatic awareness deserves a central place in psychological interventions (Ye, 2010). When students attend to bodily signals—such as the flow of breath or the release of muscular tension—they can regulate emotions through direct somatic pathways rather than through metacognitive control alone.

Despite its theoretical promise, the empirical translation of embodied cognition into structured, curriculum-based mental health interventions remains scarce (Macrine and Fugate, 2020). Most research has focused on cognitive or academic outcomes rather than on psychologically vulnerable student populations who do not meet clinical diagnostic criteria (Castro-Alonso et al., 2024). Systematic reviews of dance and movement interventions report psychological benefits, yet heterogeneity in design and measurement limits generalizability (Koch et al., 2019; Schachtler Dwarika and Haraldsen, 2023).

Quantitative research has rarely attended to students’ subjective descriptions of bodily feelings (Koch et al., 2019), while qualitative research often struggles to connect lived experience with symptom improvement (Finlay, 2011). Mixed-methods designs are therefore particularly important when bodily experience itself is a key factor (Creswell and Plano Clark, 2018). Explanatory sequential mixed-methods designs are especially suited for intervention research seeking both outcome signals and process-level understanding, as they provide contextualized interpretation of quantitative patterns through qualitative inquiry (Creswell and Plano Clark, 2018; Fetters et al., 2013).

### 1.4 Research purpose and questions

Methodological guidance consistently emphasizes that exploratory research is essential for assessing feasibility, acceptability, implementation fidelity, and preliminary outcome patterns prior to large-scale controlled trials (Leon et al., 2011; Thabane et al., 2010). Following Bowen et al. (2009), feasibility encompasses procedural (can it be delivered?), process (does it operate smoothly?), and resource (what is required?) dimensions, while acceptability refers to participants’ subjective satisfaction and perceived appropriateness. In embodied, culturally grounded movement practices conducted in real-world university environments, exploratory mixed-methods research provides the necessary foundation for generating empirically grounded hypotheses.

Accordingly, this study adopts an explanatory sequential mixed-methods exploratory design (Creswell and Plano Clark, 2018). The intervention was grounded in embodied cognition theory (Ye, 2010), with quantitative data tracking psychological and physical changes and qualitative data capturing embodied experiences in daily life (Fetters et al., 2013). It is critical to emphasize that this study was explicitly designed as exploratory rather than as an efficacy trial (Leon et al., 2011). Statistical analyses were planned as exploratory and hypothesis-generating, with effect sizes interpreted as preliminary signals rather than confirmatory evidence.

### 1.5 Research questions

Based on these objectives, this study addresses the following research questions:

*RQ1*: Does the Dao Yin class demonstrate signals of feasibility (recruitment, retention, attendance, adherence) and acceptability (satisfaction, perceived appropriateness) when delivered as a regular university course for psychologically vulnerable students?

*RQ2*: What preliminary changes occur in psychological symptoms and perceived physical status from pre- to post-intervention, and what are the associated effect sizes?

*RQ3*: How do students describe their embodied experiences of the intervention, and in what ways do these qualitative descriptions converge with or diverge from quantitative signals of change?

## 2 Methods

### 2.1 Research design

This study employed an explanatory sequential mixed-methods exploratory design. The purpose of the study was not to test treatment efficacy under controlled conditions, but to examine whether a Dao Yin-based body-oriented course delivered within a regular university setting could be implemented in a feasible and acceptable manner for psychologically vulnerable students, while also generating preliminary quantitative and qualitative signals to inform future research. This positioning is consistent with methodological guidance for pilot and exploratory studies, in which implementation processes, acceptability, and early outcome patterns are prioritized over definitive causal inference.

The quantitative component used a single-group pre-post design embedded within an existing university course. This naturalistic design was selected to preserve ecological validity and to reflect the conditions under which such body-oriented practices would most plausibly be offered in higher education. Because the course was delivered as part of routine teaching rather than as a stand-alone experimental intervention, the study was intended to observe a real educational practice in context rather than to impose a tightly controlled treatment protocol.

The qualitative component followed the quantitative data collection and was used to interpret, elaborate, and contextualize the observed pre-post patterns. Quantitative and qualitative strands were analyzed separately and then integrated during interpretation to identify areas of convergence, complementarity, and unevenness across findings. Reporting of the qualitative component was informed by the Standards for Reporting Qualitative Research (SRQR; O’Brien et al., 2014), and reporting of the mixed-methods design and integration was guided by the Good Reporting of A Mixed Methods Study framework (GRAMMS; O’Cathain et al., 2008).

The study was approved by the Ethics Committee of Taishan University (Approval No. TN20250015) and conducted in accordance with the Declaration of Helsinki. Written informed consent was obtained from all participants prior to data collection.

### 2.2 Participants and recruitment

#### 2.2.1 Course context and research integration

The study was conducted within an existing university elective course titled Traditional Chinese Body–Mind Cultivation (2 credits), offered as a general education course open to undergraduate students. The course ran across a 16-week semester, with one 90-min session per week, and formed part of the regular curriculum rather than a clinical or counseling service. The course was not created specifically for this study, and its general educational orientation remained unchanged during the research period.

A research-within-curriculum approach was adopted. In other words, the course proceeded in its usual classroom format, while students who met study eligibility criteria were invited to complete additional research assessments before and after the course, as well as optional post-course qualitative sharing. Participation in the research component was voluntary and had no effect on course enrollment, attendance requirements, or academic grading. This approach allowed the study to remain closely aligned with ordinary educational practice while preserving the integrity of the course as a public university offering.

#### 2.2.2 Screening and eligibility

**Figure 1:**
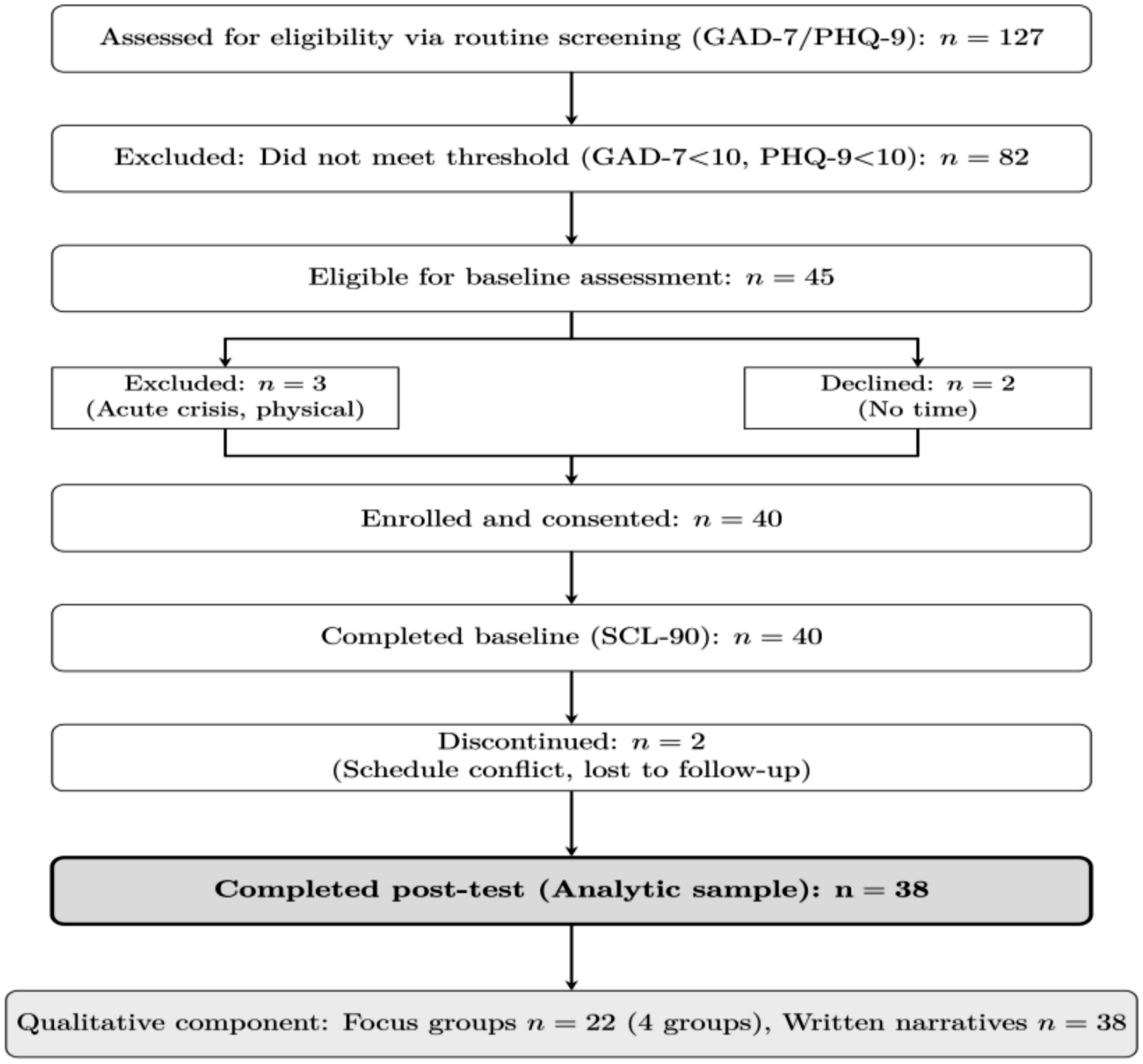
Study flow diagram showing participant recruitment and retention throughout the Dao Yin course intervention.

To identify psychologically vulnerable students without relying on diagnostic labeling, a two-stage screening procedure was implemented (see Figure 1).

Stage 1: Routine campus screening. During the semester, 2,847 students completed the university’s routine mental health screening administered through the student counseling center. Of these, 127 students (4.5%) scored above the distress threshold on the GAD-7 (≥10) or PHQ-9 (≥10) and were assessed for study eligibility. Eighty-two were excluded for subthreshold symptoms, leaving 45 eligible for baseline assessment.

Stage 2: Research eligibility verification. Research assistants (graduate students trained in study protocols) contacted the 45 eligible students via telephone to explain the study, verify course enrollment, and assess exclusion criteria. Three students were excluded (acute psychiatric crisis, *n* = 2; serious physical restriction, *n* = 1), Forty students enrolled. Two later withdrew because of course scheduling conflicts.

Inclusion criteria were: (a) current full-time undergraduate enrollment; (b) enrollment in the target elective course “Traditional Chinese Body-Mind Cultivation” during the semester of data collection; (c) elevated psychological distress defined as GAD-7 ≥10 or PHQ-9 ≥10 on routine screening, confirmed by baseline SCL-90 administration; and (d) provision of written informed consent. Exclusion criteria were limited to circumstances making movement participation unsafe: acute psychiatric crisis requiring immediate clinical intervention, serious physical conditions restricting movement (e.g., acute musculoskeletal injury), or inability to complete study procedures.

The goal was to recruit a subclinical, psychologically vulnerable sample rather than a clinically diagnosed population. Forty students enrolled and completed baseline SCL-90 assessments; 38 completed post-test (95% retention), forming the quantitative analytic sample. Participant flow is detailed in Figure 1.

#### 2.2.3 Feasibility and acceptability indicators

Because this study was designed as an exploratory classroom-based pilot rather than a controlled intervention trial, feasibility and acceptability were evaluated primarily through implementation-related indicators available within the natural course context. Feasibility was assessed in relation to course enrollment, post-enrollment retention, session attendance, completion of pre- and post-course assessments, continuity of course delivery across the semester, and the absence of serious adverse events. Acceptability was assessed in relation to continued course participation and participants’ qualitative accounts of the course as tolerable, meaningful, and useful.

As the study was embedded within an intact university public course selected through the institutional course registration system, formal trial-style implementation indicators, such as advertisement-based recruitment yield, randomization uptake, and detailed protocol deviation logs, were not applicable. Accordingly, feasibility and acceptability findings should be interpreted as naturalistic indicators of classroom implementation rather than as definitive benchmarks of intervention performance.

### 2.3 Dao Yin course context and delivery

#### 2.3.1 Positioning of the course

In the present study, Dao Yin was positioned as a culturally grounded educational movement practice rather than as psychotherapy, dance/movement therapy, or a clinical treatment approach. The course emphasized gentle movement, postural regulation, coordinated breathing, attentional settling, and awareness of bodily state. Within the university context, it was implemented as a health-promoting classroom practice intended to support body awareness and self-regulation in a non-stigmatizing educational setting. This positioning is important because the course was offered as part of regular public university teaching rather than as a therapeutic program for students with diagnosed mental disorders.

The course design was also informed by embodied cognition theory, which views cognition, emotion, and self-regulation as grounded in bodily states and actions. From this perspective, posture, movement rhythm, breath, and bodily attention are not merely secondary expressions of internal states but may actively shape subjective experience and regulation. Dao Yin was therefore relevant to the present study because it provided a body-based classroom practice through which students could engage processes of bodily awareness, attentional regulation, and gradual self-settling in an ordinary educational context.

**Figure 2:**
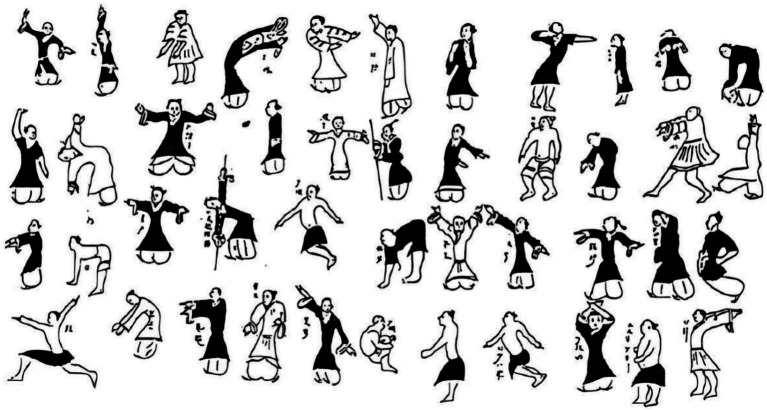
The Mawangdui Daoyin Tu (silk exercise chart excavated from the Han tombs at Mawangdui).

Historically, the movement tradition informing the course may be traced to the Mawangdui Daoyin Tu, a silk exercise chart unearthed from the Mawangdui Han tombs (see Figure 2). In contemporary China, this traditional movement heritage has been reorganized and adapted into modern Dao Yin exercise forms for educational, health-promotion, and public practice contexts. The course instructor was a professor specializing in dance therapy and a core member of the university’s Dance Therapy Research Team, with over 15 years of clinical and teaching experience in body–mind interventions. The movement routine was collaboratively developed by the research team and the instructor, drawing upon the “Mawangdui Daoyin Tu” (silk exercise chart unearthed from the Mawangdui Han tombs) as the primary historical reference. Rather than constituting a literal reconstruction of all 44 illustrated postures, the curriculum represents a pedagogical adaptation that: (1) analyzed the original chart to identify fundamental movement patterns (e.g., spinal extension, rotational stretching, and breath-movement coordination); (2) reorganized these classical forms into the five progressive units described above, tailored for beginners without prior movement training; and (3) integrated principles from embodied cognition theory to emphasize interoceptive awareness and body–mind integration during practice. While the instructor was a member of the broader dance therapy research collective, she was not directly involved in the quantitative data collection or analysis to minimize potential bias in outcome assessment.

#### 2.3.2 Overall course progression

The course was delivered across 16 weekly sessions of approximately 90 min each. Although each class followed a consistent general structure, the movement content developed progressively across the semester in five broad pedagogical units. These units should not be understood as five isolated exercises. Rather, they represented the main learning progression of the course and the recurring movement vocabulary developed over time.

Across the semester, the emphasis moved from initial settling and bodily preparation, to gentle mobilization of the spine and limbs, to more coordinated movement with breath awareness, to fuller postural opening and whole-body regulation, and finally to integrative practice and review. More specifically, Unit 1 focused on settling, standing alignment, and basic awareness of breathing and bodily tension. Unit 2 emphasized gentle stretching and mobilization of the back body and lateral body. Unit 3 introduced more coordinated turning, shifting, and breath-linked movement. Unit 4 emphasized upper-body opening, postural expansion, and smoother whole-body coordination. Unit 5 focused on integration, continuity of movement, and consolidation of self-practice strategies. The final session was used primarily for review and integration of previously learned material.

This progression was designed to remain accessible to ordinary university students while retaining the broad pedagogical characteristics of Dao Yin practice, namely regulated posture, paced movement, coordinated breath, and inward attention. Accordingly, the five units should be understood as the core pedagogical organization of the semester-long course rather than as unrelated one-off movement examples.

#### 2.3.3 Session structure

Each 90-min session followed a consistent four-phase classroom structure. First, sessions began with a brief preparatory phase involving standing regulation, gentle loosening of the body, and a gradual shift of attention toward breathing and bodily sensation. In this course, this opening phase functioned less as a conventional aerobic warm-up than as a centering and readiness practice, helping students transition from an academic classroom mode to a quieter bodily mode of attention.

Second, the main practice phase consisted of guided Dao Yin-informed movement sequences. These movements emphasized spinal mobility, upper- and lower-limb coordination, gradual extension and release, directional changes, and smooth transitions between postures. The movements used in the course were adapted for beginners and taught in a form suitable for a general university elective rather than for specialist movement training. The emphasis was placed on safe participation, repeated practice, and increasing familiarity with the rhythm and quality of movement over time, rather than on technical precision or mastery of a fixed traditional repertoire.

Third, breath awareness was integrated throughout the movement practice rather than treated as a fully separate stand-alone segment. The instructor used simple verbal prompts to encourage participants to notice breathing rhythm, muscular effort, bodily tension, and the felt quality of movement. In this way, respiration functioned as a continuous organizing thread linking posture, movement, and attention. This description more accurately reflects the embodied logic of the course than presenting breathing as an isolated exercise block.

Fourth, each session ended with a brief closing phase involving low-intensity movement, stillness, or quiet bodily observation. This phase was intended to help participants settle after practice, notice post-practice bodily and emotional changes, and leave the class in a more regulated state. In the context of the course, the closing phase functioned as a pedagogical integration period rather than a therapeutic debriefing or interpretive processing session.

#### 2.3.4 Instructor and delivery consistency

All sessions were delivered by the regular course instructor in order to maintain continuity of teaching style and classroom rhythm across the semester. The routine was organized for use with ordinary university students in a general educational setting and emphasized accessibility, gradual progression, moderate intensity, and repeated participation by students with varied movement backgrounds.

To support implementation consistency, the course followed a stable semester-long teaching outline, with each weekly session organized around the same four-phase structure described above. Although the specific movement emphasis developed progressively across the five pedagogical units, the underlying classroom logic remained consistent across the 16 weeks: centering and preparation, guided movement practice, breath-linked attentional regulation, and closing integration. This consistency helped ensure that students encountered the practice as a coherent course experience rather than as a series of unrelated activities.

At the same time, the course retained the flexibility necessary for ordinary teaching. Minor adjustments in pacing, verbal cueing, and repetition were made in response to students’ familiarity, comfort, and level of participation, but these adjustments did not alter the overall pedagogical structure or the body–mind principles guiding the course.

### 2.4 Measures

#### 2.4.1 Symptom Checklist-90

Psychological distress was assessed using the Symptom Checklist-90 (SCL-90; Derogatis, 1977), a multidimensional self-report instrument measuring the severity of recent psychological symptoms. In the present study, the SCL-90 was used to assess both overall symptom burden and variation across specific symptom domains among psychologically vulnerable university students. The scale contains 90 items covering nine primary dimensions: somatization, obsessive-compulsive symptoms, interpersonal sensitivity, depression, anxiety, hostility, phobic anxiety, paranoid ideation, and psychoticism. Items are rated on a 5-point Likert scale from 0 (“not at all”) to 4 (“extremely”). The instrument yields dimensional subscale scores as well as a Global Severity Index (GSI), defined as the mean score across all 90 items. The Chinese version of the SCL-90 has demonstrated acceptable reliability and validity in Chinese university student populations (Jin et al., 1986). In the present study, the SCL-90 total score was used as the primary quantitative indicator of overall psychological distress, while the nine dimensional scores were examined as secondary exploratory indicators of symptom-specific change. The standardized Cronbach’s alpha for the total scale in the present sample was 0.836.

#### 2.4.2 Perceived physical status

We developed a six-item self-report index to capture participants’ subjective sense of bodily comfort and everyday physical wellbeing. Consistent with the study’s focus on embodied change, perceived physical status was conceptualized as individuals’ global evaluation of their general physical condition and daily somatic discomfort.

##### 2.4.2.1 Item content and scoring

The index assessed six domains: (1) general physical condition, (2) fatigue, (3) bodily tension or stiffness, (4) sleep quality, (5) physical energy levels, and (6) the perceived impact of physical discomfort on daily functioning and mood. Items were rated on a 5-point Likert scale (1 = very poor/always interferes, 5 = excellent/never interferes). Item scores were summed to yield a total score ranging from 6 to 30, with higher total scores indicating better perceived physical status.

##### 2.4.2.2 Psychometric properties

In the present sample, the 6-item index demonstrated acceptable internal consistency (Cronbach’s *α* = 0.76 at baseline, 0.81 at post-test). Exploratory factor analysis supported a unidimensional structure explaining 58.4% of the variance, suggesting that the items captured a coherent construct of perceived physical wellbeing.

##### 2.4.2.3 Analytic positioning

This index served as a supplementary exploratory outcome to examine whether participants showed concurrent improvements in subjective physical status alongside psychological changes. As a study-specific contextual measure rather than a standalone standardized instrument, findings should be interpreted as preliminary self-perceived physical status ratings requiring validation in future research.

### 2.5 Qualitative data collection and analysis

Following completion of the course and post-test questionnaires, qualitative data were collected to explore how participants experienced the course and how they understood any changes in bodily state, emotional settling, self-awareness, or daily self-regulation. Data were gathered through focus groups and written narrative reflections using a common semi-structured prompt framework. Prompts invited participants to describe their experience of the course, perceived bodily and emotional changes, elements they found helpful or difficult, and whether anything from the course carried over into daily life.

The qualitative data were collected in Chinese. Focus groups were conducted after the quantitative phase and were facilitated by members of the research team rather than by the course instructor, in order to reduce pressure for socially desirable responses. The instructor was not present during the group discussions. Written narratives were collected separately to allow participants to express experiences that they might not wish to share in a group setting.

Data were analyzed using reflexive thematic analysis. The analytic process involved repeated reading of transcripts and written responses, initial coding of meaningful segments, development of candidate patterns across cases, and iterative refinement of themes through ongoing interpretive discussion within the research team. In keeping with reflexive thematic analysis, the emphasis was not on achieving coder agreement as a positivist reliability exercise, but on developing a coherent and conceptually meaningful interpretation of participants’ accounts. The researchers also considered how their own professional backgrounds in movement, education, and student wellbeing might shape the reading of the data.

### 2.6 Data analysis and integration

Quantitative data were analyzed using descriptive statistics and paired-samples comparisons between baseline and post-course scores. Change scores were inspected for approximate distributional reasonableness prior to inferential analysis. For repeated-measures comparisons, effect sizes were reported to aid interpretation of the magnitude of change. For paired-samples analyses, Cohen’s *d* was calculated as the mean pre-post difference divided by the standard deviation of the difference scores. Because this was an exploratory pilot study with a modest sample size, statistical analyses were interpreted as preliminary and hypothesis-generating. No formal correction for multiple comparisons was applied to the SCL-90 subscale analyses; accordingly, subscale-level findings were treated as descriptive and should be interpreted with caution.

Quantitative and qualitative findings were integrated during the interpretation stage through comparison of the overall direction, emphasis, and texture of the results. The aim of integration was not merely to confirm quantitative change with qualitative illustration, but to examine how participants’ embodied accounts helped explain, deepen, or nuance the pre-post patterns observed in the questionnaire data. Areas of convergence, complementarity, and unevenness were therefore considered in the mixed-methods interpretation.

## 3 Results

### 3.1 Sample characteristics and feasibility indicators

Figure 1 presents the CONSORT flow diagram. Of 127 students assessed through routine campus screening, 45 met eligibility criteria for baseline assessment. Forty students enrolled in the course and provided informed consent, of whom 38 (95.0%) completed both pre- and post-intervention assessments. Two students withdrew after enrollment because of course scheduling conflicts.

#### 3.1.1 Sample characteristics

The analytic sample comprised 38 undergraduate students. Participants represented diverse academic disciplines including humanities (*n* = 14), science and engineering (*n* = 16), and arts-related majors (*n* = 8). Baseline SCL-90 scores indicated elevated psychological distress across multiple dimensions (Table 1), consistent with the inclusion criteria targeting psychologically vulnerable students.

**Table 1:** Demographic characteristics and baseline psychological symptoms (*n* = 38).

Variable	Category/M (SD)	*n*/value	%
Sex	Male	12	31.6
	Female	26	68.4
Age	M ± SD	19.97 ± 0.49	—
Academic discipline	Humanities	14	36.8
	Science/Engineering	16	42.1
	Arts	8	21.1
Baseline SCL-90			
Total score	M (SD)	142.58 (39.39)	—
Somatization	M (SD)	19.29 (6.72)	—
Obsessive-compulsive	M (SD)	20.29 (6.34)	—
Depression	M (SD)	20.82 (8.18)	—
Anxiety	M (SD)	16.68 (8.37)	—
Physical status	M (SD)	12.18 (3.76)	—

Feasibility outcomes are summarized in Table 2. Course retention was high, with 38 of the 40 enrolled students completing the course and post-course assessment (95.0%). Attendance was consistent (M = 14.2 of 16 sessions, SD = 1.3; range = 11–16), and complete assessment data were obtained for all completers. Participant burden was low, with questionnaire completion requiring less than 25 min, and no adverse events were recorded. Acceptability was reflected in students’ continued participation in the course and in qualitative accounts describing the course as useful, meaningful, and manageable.

**Table 2:** Feasibility and acceptability indicators (*n* = 40 enrolled).

Domain	Indicator	Result
Course implementation	Enrolled students	40
Course implementation	Post-enrollment withdrawal	2 (course scheduling conflict)
Course implementation	Retention rate	95.0% (38/40)
Process	Mean attendance (SD)	14.2 (1.3) of 16 sessions
Process	Assessment completion	100% (38/38 completers)
Resource	Participant burden	<25 min per assessment
Resource	Adverse events	0
Acceptability	Continued course participation	38/40 completed the course
Acceptability	Qualitative acceptability	Participants described the course as useful, meaningful, and manageable

### 3.2 Preliminary quantitative outcomes

#### 3.2.1 Psychological symptoms

Paired-samples analyses indicated reductions in overall psychological symptom burden from pre- to post-intervention (Table 3). The SCL-90 total score decreased from baseline (M = 142.58, SD = 39.39) to post-intervention (M = 122.34, SD = 27.80), yielding a mean difference of 20.24 [95% CI (13.71, 26.77)], *t*(37) = 6.30, *p* < 0.001, *d* = 1.02 [95% CI (0.68, 1.36)].

**Table 3:** Pre-post changes in SCL-90 and physical status (*n* = 38).

Measure	Pre M (SD)	Post M (SD)	Mean diff. (95% CI)	*t*(37)	*p*	Cohen’s *d* (95% CI)
SCL-90 total	142.58 (39.39)	122.34 (27.80)	20.24 (13.71, 26.77)	6.3	<0.001	1.02 (0.68, 1.36)
Somatization	19.29 (6.72)	16.00 (3.42)	3.29 (1.65, 4.93)	4.06	<0.001	0.66 (0.33, 0.98)
Obsessive-compulsive	20.29 (6.34)	17.11 (4.78)	3.18 (1.60, 4.76)	4.08	<0.001	0.66 (0.33, 0.99)
Interpersonal sensitivity	14.08 (5.30)	12.55 (3.87)	1.53 (0.77, 2.29)	4.09	<0.001	0.66 (0.33, 0.99)
Depression	20.82 (8.18)	17.08 (5.06)	3.74 (2.16, 5.32)	4.79	<0.001	0.78 (0.44, 1.11)
Anxiety	16.68 (8.37)	13.16 (3.94)	3.52 (1.45, 5.59)	3.46	0.001	0.56 (0.23, 0.88)
Hostility	8.76 (2.83)	7.68 (2.79)	1.08 (0.25, 1.91)	2.64	0.012	0.43 (0.10, 0.75)
Phobic anxiety	9.55 (3.50)	8.82 (2.68)	0.73 (−0.10, 1.56)	1.8	0.08	0.29 (−0.03, 0.61)
Paranoid ideation	8.84 (2.78)	7.84 (1.98)	1.00 (0.38, 1.62)	3.29	0.002	0.53 (0.20, 0.86)
Psychoticism	13.34 (4.14)	12.63 (3.32)	0.71 (−0.15, 1.57)	1.69	0.1	0.27 (−0.05, 0.60)
Physical status	12.18 (3.76)	15.42 (3.21)	3.24 (2.12, 4.36)	5.14	<0.001	0.83 (0.49, 1.17)

Perceived physical status scored such that higher values indicate better physical wellbeing.

##### 3.2.1.1 Note on interpretation

As an exploratory pilot study without a control condition, these effect sizes reflect pre-post change in a single group and likely encompass multiple non-specific factors (e.g., expectancy, regression to the mean, natural course of symptoms) alongside any intervention-specific effects. Results should be interpreted as preliminary signals requiring confirmation in controlled trials.

At the dimensional level, statistically significant reductions were observed for somatization, obsessive-compulsive symptoms, interpersonal sensitivity, depression, anxiety, hostility, and paranoid ideation (all *p* < 0.05, Table 3). Effect sizes ranged from medium to large (*d* = 0.43 to 1.02). However, phobic anxiety and psychoticism showed non-significant changes (*p* = 0.08 and *p* = 0.10, respectively).

##### 3.2.1.2 Multiple comparisons

Given the exploratory nature of this pilot study and the risk of Type II error with a modest sample, we did not apply Bonferroni correction to the nine subscale comparisons. Subscale analyses should be interpreted as descriptive and hypothesis-generating rather than confirmatory.

#### 3.2.2 Perceived physical status

The Perceived Physical Status Index showed significant improvement from pre- (M = 12.18, SD = 3.76) to post-intervention (M = 15.42, SD = 3.21), *t*(37) = 5.14, *p* < 0.001, *d* = 0.83 [95% CI (0.49, 1.17)]. This change indicates improved self-reported physical comfort, reduced fatigue, and better sleep quality following the course.

### 3.3 Qualitative findings

#### 3.3.1 Data source and analytic overview

Four semi-structured focus groups were conducted with 22 participants (M = 5.5 per group; duration: 65–85 min), supplemented by written reflective narratives from all 38 participants. Reflexive thematic analysis yielded four interconnected themes describing participants’ embodied experiences: (1) Bodily Loosening and Physical Relief, (2) Emotional Settling, (3) Body–Mind Awareness, and (4) Everyday Self-Regulation (Table 4).

**Table 4:** Joint display of quantitative outcomes and qualitative themes.

Quantitative pattern	Qualitative theme	Integration type	Interpretation
↓ SCL-90 total score (large effect)	Bodily loosening	Convergence	Symptom reduction accompanied by experiential relief of physical tension
↓ Somatization (*d* = 0.66)	Heightened body awareness (in some)	Divergence	Quantified symptom decrease co-occurred with *increased* sensitivity to bodily signals, suggesting shift from distress to mindful awareness
↓ Depression, anxiety	Emotional settling	Convergence	Mood improvements reflected in subjective reports of stability and reduced reactivity
↑ Physical status index	Everyday self-regulation	Convergence	Quantified physical improvement explained by qualitative reports of strategy transfer
↔ Phobic anxiety (ns)	Minimal qualitative reference	Divergence	Intervention less relevant for specific phobic symptoms; focus on general stress rather than specific fears
↔ Psychoticism (ns)	Absent in qualitative data	Divergence	No evidence of effect on psychotic symptoms; intervention targets stress-related

↓, significant reduction; ↑, significant improvement; ↔, non-significant change; ns, not significant.

#### 3.3.2 Theme 1: Bodily loosening and physical relief

Participants consistently reported reductions in muscular tension and physical discomfort. Exemplar quotations included: “After the course, the muscles in my body clearly feel more relaxed, my sleep quality has improved” and “My body feels lighter and my brain can relax.” This theme aligns with quantitative reductions in the SCL-90 Somatization subscale.

#### 3.3.3 Theme 2: Emotional settling

Participants described increased emotional stability and reduced reactivity. One participant noted: “My mood has improved and become more stable; I no longer get so angry that I end up in big quarrels.” Another stated: “I don’t overthink as much, and when I face difficulties I feel calmer than before.”

#### 3.3.4 Theme 3: Body–mind awareness

Participants reported enhanced recognition of connections between bodily states and emotional experiences. “I feel that my body and mind are being adjusted at the same time” and references to “Form–spirit unity” (形神合一) illustrated integrated body–mind experiences.

#### 3.3.5 Theme 4: Everyday self-regulation

Participants described transferring learned practices into daily life: “When I feel anxious, I do deep breathing and a short meditation” and “Before class I sometimes felt gloomy, but after the session my mood feels much more relaxed.”

### 3.4 Mixed-methods integration

#### 3.4.1 Areas of convergence

Substantial convergence emerged between quantitative symptom reductions and qualitative experiential accounts. Decreases in SCL-90 Total Score and Somatization aligned with Theme 1 (bodily loosening), wherein participants subjectively reported reduced tension and improved sleep. Similarly, quantitative reductions in Depression and Anxiety corresponded with Theme 2 (emotional settling), suggesting that symptom improvement was accompanied by experientially meaningful changes in emotional regulation.

Improvements in the Physical Status Index converged with Theme 4 (everyday self-regulation). Participants’ qualitative accounts of applying breathing and stretching techniques outside class provided contextual explanation for the observed quantitative gains in physical comfort.

#### 3.4.2 Areas of divergence and nuanced understanding

The integration also revealed important divergences that delimited the boundaries of observed effects. Phobic Anxiety and Psychoticism showed non-significant quantitative changes (*p* = 0.08 and 0.10, respectively), with limited qualitative reference to these specific symptom domains. This divergence suggests that the Dao Yin intervention, focusing on general somatic awareness and stress reduction, may have less direct relevance for specific phobic fears or attenuated psychotic symptoms compared to general anxiety or depressive affect.

A second divergence emerged between somatization scores and body awareness descriptions. While somatization showed significant reduction (*d* = 0.66), some participants in focus groups reported heightened somatic awareness (e.g., “I became more sensitive to subtle tensions in my body”). This apparent contradiction resolves when considering that increased interoceptive sensitivity (awareness) may accompany decreased symptomatic distress (tension), reflecting a shift from dysfunctional somatic preoccupation to adaptive bodily awareness—a nuance quantitative scores alone could not capture.

##### 3.4.2.1 Synthesis

The mixed-methods integration indicates that pre-post quantitative changes were broadly consistent with participants’ embodied experiences, while qualitative data elaborated how changes occurred (e.g., through daily self-regulation practices) and identified boundaries (e.g., limited effect on phobic symptoms). These integrated findings provide a coherent account of preliminary outcome signals within an exploratory framework, highlighting both the potential and the limitations of classroom-based Dao Yin for psychologically vulnerable students.

## 4 Discussion

### 4.1 Summary of main findings

This exploratory study examined whether a Dao Yin-based movement course could be feasibly delivered within a regular university curriculum and generate preliminary signals of effect for psychologically vulnerable students. Three primary findings emerged. First, feasibility and acceptability indicators were favorable: the course achieved 95% retention, high attendance rates (M = 14.2/16 sessions), and sustained student participation, suggesting that classroom-based Dao Yin is viable within standard higher education structures. Second, quantitative analyses revealed pre-post improvements in overall psychological distress (SCL-90 total score: *d* = 1.02) and perceived physical status (*d* = 0.83), though these effect sizes likely reflect non-specific factors including expectancy and natural course effects rather than intervention-specific efficacy. Third, qualitative findings illuminated experiential mechanisms, with participants reporting enhanced body–mind awareness, emotional regulation strategies, and integration of practices into daily life.

It is critical to emphasize that these findings are exploratory and hypothesis-generating, consistent with the pilot study design (Leon et al., 2011; Thabane et al., 2010). Without a control group, we cannot attribute observed changes solely to the intervention, nor can we estimate the magnitude of effects independent of confounding variables such as regression to the mean or seasonal variation in student stress. Rather, this study establishes procedural feasibility and generates contextualized hypotheses regarding potential mechanisms—namely, that Dao Yin may operate through enhanced interoceptive awareness and somatic self-regulation—warranting testing in subsequent controlled trials.

### 4.2 Integration of quantitative and qualitative findings: convergence, divergence, and mechanistic insights

The explanatory sequential mixed-methods design permitted systematic examination of how quantitative outcome patterns aligned with or diverged from participants’ lived experiences (Fetters et al., 2013). The use of a joint display and explicit narrative comparison also followed GRAMMS guidance for transparent reporting of where and how integration occurred.

Areas of convergence provided cross-methodological validation. The large reduction in SCL-90 somatization scores (*d* = 0.66) converged with qualitative accounts of “bodily loosening” and reduced muscular tension, suggesting that quantified symptom relief corresponded to experientially meaningful somatic changes. Similarly, improvements in perceived physical status aligned with qualitative descriptions of adopting breathing and stretching practices during daily stressors, indicating behavioral transfer beyond the classroom context.

Areas of divergence yielded nuanced insights that quantitative data alone could not provide. While phobic anxiety showed non-significant quantitative change (*p* = 0.08), qualitative data revealed minimal participant concern regarding specific phobias, suggesting that the intervention’s focus on general stress reduction and somatic awareness may be less relevant for circumscribed anxiety disorders requiring exposure-based approaches. This divergence appropriately delimits the intervention’s potential scope.

More intriguingly, expansion occurred regarding the relationship between somatic awareness and distress. Quantitative somatization scores decreased, yet qualitative accounts described heightened sensitivity to bodily signals (e.g., “I became more aware of tension in my shoulders”). Rather than contradiction, this pattern suggests a shift from dysfunctional somatic preoccupation (anxiety-driven hypervigilance associated with distress) to adaptive interoceptive awareness (mindful attention facilitating regulation)—a distinction that symptom scales alone cannot capture (Mehling et al., 2012). This expansion generates the hypothesis that Dao Yin may recalibrate the quality of body awareness rather than merely reducing somatic complaints.

### 4.3 Theoretical interpretation: embodied cognition and somatic self-regulation

These findings can be interpreted through the lens of embodied cognition theory (Ye, 2010; Macrine and Fugate, 2020), which posits that cognitive and affective processes are constituted by bodily states rather than merely correlated with them. From this perspective, Dao Yin practices may facilitate psychological well-being through three interrelated mechanisms.

First, postural and movement-based regulation: Dao Yin’s emphasis on spinal elongation, gentle twisting, and weight distribution (the “three adjustments” of body, breath, and mind) may directly influence affective state via bidirectional body–brain feedback loops. Participants’ reports of “feeling lighter” and “more stable” align with evidence that expansive, aligned postures correlate with reduced cortisol and increased testosterone (Carney et al., 2010), though physiological measurement would be required to confirm such mechanisms.

Second, interoceptive recalibration: The cultivation of sustained attention to bodily sensations (e.g., breath rhythm, muscular tension, qi flow) may enhance interoceptive accuracy—the ability to detect and interpret physiological signals—while reducing interoceptive anxiety (fear of bodily sensations). This distinguishes Dao Yin from purely physical exercise; the qualitative emphasis on “harmonizing qi and blood” suggests participants developed interpretive frameworks for somatic experiences that normalized bodily variations rather than catastrophizing them.

Third, contextual embedding of regulation skills: Qualitative accounts of using breathing techniques “before exams” or during anxiety episodes indicate that participants internalized somatic self-regulation as transferable skills. This supports the theoretical prediction that embodied practices can generate “somatic markers”—gut feelings that guide adaptive decision-making and emotional regulation in daily life (Damasio, 2000).

However, as we measured neither physiological markers (e.g., heart rate variability, cortisol) nor specific mediating variables (e.g., interoceptive accuracy scales), these mechanisms remain hypothetical and require direct testing in future research.

### 4.4 Situating findings within mind–body intervention literature

The observed effect sizes (*d* = 0.56–1.02 for primary outcomes) are consistent with meta-analytic findings for movement-based interventions including yoga, tai chi, and dance movement therapy (Cramer et al., 2013; Koch et al., 2019). However, prior research has predominantly examined these practices in clinical or elderly populations, with limited attention to subclinical, psychologically vulnerable students—a gap this study addresses.

Notably, Dao Yin shares core characteristics with tai chi and qigong (breath-movement coordination, mindfulness, traditional Chinese medical theory) yet differs in its specific emphasis on meridian-based stretching and “form-spirit unity” (形神合一). The qualitative prominence of “qi and blood harmonization” discourse suggests that participants engaged culturally specific explanatory models, which may enhance placebo effects or therapeutic alliance through cultural congruence (Ryder et al., 2008). Future comparative research should examine whether culturally grounded practices like Dao Yin offer advantages over generic mindfulness or exercise programs for Chinese students, potentially through enhanced credibility or cultural resonance.

### 4.5 Strengths

This study offers several methodological strengths. The 16-week duration exceeds that of most pilot studies, allowing examination of sustained engagement and progressive skill development. The mixed-methods integration generated mechanistic insights unavailable through either method alone, particularly regarding the somatic awareness-distress relationship. Ecological validity was enhanced by embedding the intervention within an existing university course rather than an artificial laboratory setting, ensuring findings reflect real-world implementation conditions. Finally, the absence of adverse events and high retention rates support the safety and acceptability of classroom-based Dao Yin for vulnerable students.

### 4.6 Limitations

Several limitations constrain interpretation and generalizability. Most critically, the single-group pre-post design precludes causal inference. Observed changes may reflect maturation, seasonal effects (semester progression), regression to the mean, or expectancy effects rather than intervention-specific mechanisms (Leon et al., 2011). The lack of an active control group (e.g., aerobic exercise or relaxation training) prevents estimation of specific versus non-specific effects.

Instructor effects represent a significant unmeasured variable. Because a single instructor delivered all sessions, observed outcomes may reflect the instructor’s specific charisma, teaching style, or relational warmth rather than the Dao Yin protocol itself (Koch et al., 2019). Although fidelity monitoring indicated high protocol adherence (96.3%), the “person effect” cannot be disentangled from the “practice effect” in this design. Future research should employ multiple instructors or control for instructor characteristics.

Self-report bias may have inflated observed effects, particularly given the absence of blinding. Participants aware of the study’s hypotheses may have provided socially desirable responses on the SCL-90. The reliance on a researcher-developed physical status index (rather than validated objective measures like actigraphy or medical examination) limits confidence in physical health claims.

Sample limitations include the small size (*n* = 38), limited demographic diversity (predominantly female, single university), and potential selection bias—students volunteering for a body-oriented course may possess pre-existing positive attitudes toward somatic practices, limiting generalizability to skeptical or physically avoidant individuals. Finally, the absence of longitudinal follow-up precludes examination of sustained effects or skill maintenance beyond the course duration.

### 4.7 Implications for future research and practice

Research priorities include randomized controlled trials with active control conditions (e.g., aerobic exercise, psychoeducation) to isolate specific effects, and mechanistic studies incorporating physiological measures (autonomic function, inflammatory markers) and validated interoception scales to test the embodied cognition hypotheses generated here. Future research should also examine optimal dosing—whether benefits require the full 16-week protocol or whether shorter interventions suffice—and moderators of response (e.g., baseline body awareness, cultural identification).

Practical implications are immediate. Given rising student mental health concerns and barriers to traditional counseling (stigma, service gaps), classroom-based Dao Yin offers a low-threshold, non-stigmatizing, culturally congruent prevention approach embeddable within existing curriculum structures. Educational policymakers might consider accrediting such courses as mental health promotion electives, while clinicians could explore Dao Yin as complementary support for students with subclinical distress. However, such integration should proceed cautiously, recognizing that this study establishes feasibility and preliminary signals—not efficacy warranting clinical recommendation as a standalone treatment.

## 5 Conclusion

This exploratory study demonstrates that a 16-week Dao Yin course is feasible and acceptable for psychologically vulnerable university students, generating preliminary signals of improved psychological and physical well-being. Mixed-methods integration suggests potential mechanisms involving enhanced interoceptive awareness and somatic self-regulation, while also delineating boundaries (e.g., limited relevance for phobic anxiety). These findings provide empirical grounding for subsequent controlled trials examining the efficacy, mechanisms, and scalability of culturally grounded, body-oriented mental health promotion in higher education settings.

## Data Availability

The datasets presented in this study can be found in online repositories. The names of the repository/repositories and accession number(s) can be found in the article/Supplementary material.

## References

[ref1] Auerbach R. P. Mortier P. Bruffaerts R. Alonso J. Benjet C. Cuijpers P. . (2018). WHO world mental health surveys international college student project: prevalence and distribution of mental disorders. J. Abnorm. Psychol. 127, 623–638. doi: 10.1037/abn0000362, 30211576 PMC6193834

[ref2] Bowen D. J. Kreuter M. Spring B. Cofta-Woerpel L. Linnan L. Weiner D. . (2009). How we design feasibility studies. Am. J. Prev. Med. 36, 452–457. doi: 10.1016/j.amepre.2009.02.002, 19362699 PMC2859314

[ref3] Carney D. R. Cuddy A. J. C. Yap A. J. (2010). Power posing: brief nonverbal displays affect neuroendocrine levels and risk tolerance. Psychol. Sci. 21, 1363–1368. doi: 10.1177/0956797610383437, 20855902

[ref4] Castro-Alonso J. C. Wong M. Adesope O. O. Ayres P. Paas F. (2024). Embodied learning in education: a meta-analysis. Educ. Psychol. Rev. 36:98. doi: 10.1007/s10648-024-09823-4

[ref5] Chen X. Liu J. Wang Y. (2022). Mental health status and associated factors among Chinese university students during the COVID-19 pandemic. Front. Psychol. 13:823498. doi: 10.3389/fpsyg.2022.823498 PMC9608563 36312111

[ref9006] Chen Y. M. Zhang Y. L. Yu G. L. (2022). A meta-analysis of the detection rate of mental health problems among college students in mainland China from 2010 to 2020. Adv. Psychol. Sci. 30, 991–1004. doi: 10.3724/SP.J.1042.2022.00991

[ref6] Cramer H. Lauche R. Langhorst J. Dobos G. (2013). Yoga for depression: a systematic review and meta-analysis. Depress. Anxiety 30, 1068–1083. doi: 10.1002/da.22166, 23922209

[ref7] Creswell J. W. Plano Clark V. L. (2018). Designing and Conducting Mixed Methods Research. 3rd Edn Thousand Oaks, CA: SAGE.

[ref8] Damasio A. R. (2000). The Feeling of What Happens: Body and Emotion in the Making of Consciousness. New York, NY: Vintage Books.

[ref9] Derogatis L. R. (1977). SCL-90-R, Administration, Scoring & Procedures Manual—I for the R(evised) Version. Baltimore, MA: John Hopkins University School of Medicine.

[ref10] Fetters M. D. Curry L. A. Creswell J. W. (2013). Achieving integration in mixed methods designs: principles and practices. Health Serv. Res. 48, 2134–2156. doi: 10.1111/1475-6773.12117, 24279835 PMC4097839

[ref11] Finlay L. (2011). Phenomenology for Therapists: Researching the Lived World. Hoboken, NJ: Wiley-Blackwell.

[ref001] Gao L. Xie Y. Jia C. Wang W. (2020). Prevalence of depression among Chinese university students: A systematic review and meta-analysis. Sci. Rep. 10:15897. doi: 10.1038/s41598-020-72998-1 32985593 PMC7522998

[ref12] Jahnke R. Larkey L. Rogers C. Etnier J. Lin F. (2010). A comprehensive review of health benefits of qigong and tai chi. Am. J. Health Promot. 24, e1–e25. doi: 10.4278/ajhp.081013-LIT-248, 20594090 PMC3085832

[ref9009] Jin H. Wu W. Zhang M. (1986). Preliminary analysis of SCL-90 evaluation results in Chinese normal subjects. Chinese Journal of Nervous and Mental Diseases. 12, 260–263.

[ref13] Koch S. C. Riege R. F. F. Tisborn K. Biondo J. Martin L. Beelmann A. (2019). Effects of dance movement therapy and dance on health-related psychological outcomes: a meta-analysis. Arts Psychother. 63:101607. doi: 10.1016/j.aip.2019.101607 PMC6710484 31481910

[ref14] Leon A. C. Davis L. L. Kraemer H. C. (2011). The role and interpretation of pilot studies in clinical research. J. Psychiatr. Res. 45, 626–629. doi: 10.1016/j.jpsychires.2010.10.008, 21035130 PMC3081994

[ref9010] Li L. Wang Y. Y. Wang S. B. Zhang L. Li L. Xu D. D. . (2018). Prevalence of sleep disturbances in Chinese university students: a comprehensive meta-analysis. J. Sleep Res. 27:e12648. doi: 10.1111/jsr.12648 29383787

[ref002] Liu X. Clark J. Siskind D. Williams G. M. Byrne G. Yang J. L. . (2021). The effects of Tai Chi and Qigong exercise on psychological status in adolescents: A systematic review and meta-analysis. Front. Psychol. 12:746975. doi: 10.3389/fpsyg.2021.746975 34899487 PMC8652254

[ref15] Ma Y. Liu Y. (2020). Research on the health benefits of traditional Chinese Dao Yin exercise. J. Sport Health Sci. 10, 360–367. doi: 10.1016/j.jshs.2019.08.003, 33993922 PMC8167318

[ref16] Macrine S. L. Fugate J. M. B. (2020). Embodied cognition: a new perspective on learning and teaching. Educ. Psychol. Rev. 32, 357–376. doi: 10.1007/s10648-019-09501-3

[ref17] Mehling W. E. Price C. Daubenmier J. J. Acree M. Bartmess E. Stewart A. (2012). The multidimensional assessment of interoceptive awareness (MAIA). PLoS One 7:e48230. doi: 10.1371/journal.pone.0048230, 23133619 PMC3486814

[ref9005] Ning X. Wong J. P. H. Huang S. Fu Y. Gong X. Zhang L. . (2022). Chinese university students’ perspectives on help-seeking and mental health counseling. Int. J. Environ. Res. Public Health. 19:8259. doi: 10.3390/ijerph19148259 35886103 PMC9323838

[ref18] O’Brien B. C. Harris I. B. Beckman T. J. Reed D. A. Cook D. A. (2014). Standards for reporting qualitative research: a synthesis of recommendations. Acad. Med. 89, 1245–1251. doi: 10.1097/ACM.0000000000000388 24979285

[ref19] O’Cathain A. Murphy E. Nicholl J. (2008). The quality of mixed methods studies in health services research. J. Health Serv. Res. Policy 13, 92–98. doi: 10.1258/jhsrp.2007.007074 18416914

[ref20] Pan Y. Li Y. Zhang Q. (2023). Help-seeking behavior and mental health among college students: the mediating role of social support. Int. J. Environ. Res. Public Health. 20:4123. doi: 10.3390/ijerph20054123, 36901133 PMC10001502

[ref21] Ryder A. G. Yang J. Zhu X. Yao S. Yi J. Heine S. J. . (2008). The cultural shaping of depression: somatic symptoms in China, psychological symptoms in North America? J. Abnorm. Psychol. 117, 300–313. doi: 10.1037/0021-843X.117.2.300, 18489206

[ref22] Schachtler Dwarika M. E. Haraldsen I. R. (2023). Dance movement therapy for mental health: a scoping review. Front. Psychol. 14:1123456. doi: 10.3389/fpsyg.2023.1123456 PMC10035338 36968742

[ref9008] Su D. Ye H. Li J. (2013). Conceptual representation from the perspective of embodied cognition: evidence from Chinese idiom comprehension. Acta Psychol. Sin. 45, 934–944. doi: 10.3724/SP.J.1041.2013.00934

[ref202] Sun C. Zhu Z. Zhang P. Wang L. Zhang Q. Guo Y. . (2024). Exploring the interconnections of anxiety, depression, sleep problems and health-promoting lifestyles among Chinese university students: a comprehensive network approach. Front. Psych. 15:1402680. doi: 10.3389/fpsyt.2024.1402680 PMC11284064 39077626

[ref23] Thabane L. Ma J. Chu R. Cheng J. Ismaila A. Rios L. P. . (2010). A tutorial on pilot studies: the what, why and how. BMC Med. Res. Methodol. 10:1. doi: 10.1186/1471-2288-10-1, 20053272 PMC2824145

[ref24] Wang C. Bannuru R. Ramel J. Kupelnick B. Scott T. Schmid C. H. (2010). Tai Chi on psychological well-being: systematic review and meta-analysis. BMC Complement. Altern. Med. 10:23. doi: 10.1186/1472-6882-10-23, 20492638 PMC2893078

[ref101] Wang X. Liu Q. (2022). Prevalence of anxiety symptoms among Chinese university students amid the COVID-19 pandemic: a systematic review and meta-analysis. Heliyon 8:e10117. doi: 10.1016/j.heliyon.2022.e10117 35965987 PMC9364719

[ref25] Wang Z. Liu H. Chen Y. (2024a). Mental health trajectories of Chinese university students in the post-pandemic era. Asian J. Psychiatry 87:103646. doi: 10.1016/j.ajp.2023.103646

[ref004] Wang Z. Li X. Xu H. Zhang T. (2024b). Risk and protective factors of suicidal tendencies among freshmen in China revealed by a hierarchical regression model. Eur. Child Adolesc. Psychiatry 33, 3043–3053. doi: 10.1007/s00787-024-02370-5 38324038

[ref9003] Xu Y. Li Y. Zhang Q. (2022). Association between sleep quality and psychological symptoms: a cross-sectional survey of Chinese university students performed during the COVID-19 pandemic. Front. Psych. 13:1131176. doi: 10.3389/fpsyt.2023.1131176 PMC10227667 37260956

[ref9007] Ye H. S. (2010). Embodied cognition: a new orientation in cognitive psychology. Adv. Psychol. Sci. 18, 705–710.

[ref003] Yu Y. She R. Luo S. Xin M. Li L. Wang S. . (2021). Factors influencing depression and mental distress related to COVID-19 among university students in China: Online cross-sectional mediation study. JMIR Ment. Health. 8:e22705. doi: 10.2196/22705 33616541 PMC7901598

[ref26] Zhang L. Chen X. (2018). Dao Yin exercise and its application in health promotion: a review. Chin. J. Integr. Med. 24, 471–476. doi: 10.1007/s11655-017-2845-3

[ref27] Zhou Y. Li X. Zhang Y. (2019). Effects of Baduanjin Dao yin exercise on physiological and psychological health in elderly patients with chronic diseases. Evid. Based Complement. Alternat. Med. 5843136. doi: 10.1155/2019/5843136

